# Okadaic Acid Toxin at Sublethal Dose Produced Cell Proliferation in Gastric and Colon Epithelial Cell Lines

**DOI:** 10.3390/md11124751

**Published:** 2013-12-02

**Authors:** Miguel del Campo, Héctor Toledo, Néstor Lagos

**Affiliations:** 1 Laboratory of Membrane Biochemistry, Program of Physiology and Biophysics, Faculty of Medicine, University of Chile, Independencia 1027, Correo 7, Santiago 8380000, Chile; E-Mail: Miguel.delcampo@fucited.cl; 2 Laboratory of Molecular Microbiology, Program of Cellular and Molecular Biology, Faculty of Medicine, University of Chile, Independencia 1027, Correo 7, Santiago 8380000, Chile; E-Mail: htoledo@med.uchile.cl

**Keywords:** okadaic acid, gastric cancer, colon cancer, PP2A, proliferation

## Abstract

The aim of this study was to analyze the effect of Okadaic Acid (OA) on the proliferation of gastric and colon epithelial cells, the main target tissues of the toxin. We hypothesized that OA, at sublethal doses, activates multiple signaling pathways, such as Erk and Akt, through the inhibition of PP2A. To demonstrate this, we carried out curves of doses and time response against OA in AGS, MKN-45 and Caco 2 cell lines, and found an increase in the cell proliferation at sublethal doses, at 24 h or 48 h exposure. Indeed, cells can withstand high concentrations of the toxin at 4 h exposure, the time chosen considering the maximum time before total gastric emptying. We have proved that this increased proliferation is due to an overexpression of Cyclin B, a cyclin that promotes the passage from G2 to mitosis. In addition, we have demonstrated that OA induces activation of Akt and Erk in the three cells lines, showing that OA can activate pathways involved in oncogenesis. In conclusion, this study contributes to the knowledge about the possible effects of chronic OA consumption.

## 1. Introduction

Okadaic acid (OA) is the principal component of diarrhetic shellfish poisoning (DSP) toxins [1]. The symptomatology of DSP is developed within 30 min to 6 h after contaminated bivalve consumption and includes diarrhea (60%), nausea (46%), vomiting (31%), and abdominal pain (77%). In addition, it generates loss of epithelial integrity, erosion and hypersecretion in the intestines and increases the paracellular permeability. If the level of intoxication is mild, the syndrome evolves favorably toward total recovery in two to three days [2,3]. While there are no records of death, these toxins have been widely described as tumor promoters [4]. In fact, it has been speculated that consumption of seafood in areas where red tide is endemic increases the risk of gastro-intestinal cancer [5]. Currently, European standard maximum levels for total DSP toxins, cannot exceed 160 μg OA equivalent/kg shellfish meat [1]. Therefore, it is permitted to consume DST in chronically at low concentrations.

OA is a potent inhibitor against Ser/Thr Protein Phosphatase 2A (PP2A) and Protein Phosphatase 1 (PP1), with an IC50 of 0.1–1 ng/mL for PP2A and 100-fold higher IC50 for PP1 [6]. The PP2A plays a key role in the regulation of major cell metabolic pathways, such as translation, transcription and control of transition from G2 to the M phase of the cell cycle; it is a tumor suppressor protein and a positive regulator of apoptosis [7]. Through the inhibition of PP2A, OA generates cellular hyperphosphorylation, which can activate multiple signaling pathways, including the expression and secretion of inflammatory agents that can act as endogenous tumor promoters such as TNF-α [8]. Experimentally, it has been demonstrated that repeated exposure of OA induces tumor formation in mouse skin, glandular stomach and rat liver [4]. While OA has been reported to increase cell proliferation [9,10], it is unknown whether it has the same effect on the gastric epithelium, the main target following oral administration of the toxin after acute doses [11,12,13]. Indeed, this effect is contradictory to its broad description as toxic agent, able to arrest the mitotic cycle, form DNA adducts and activate Caspases 3, 8 and 9, in many cell lines [14,15,16,17].

We hypothesize that the dual effect of OA is dose and time exposure dependent and that it activates cell proliferation signaling at sublethal doses. For this, the effect of OA on the viability of two models of gastric epithelium (AGS and MKN-45) and colon epithelium (Caco 2) was analyzed, studying the expression of Cyclin B1, a cyclin that promotes the passage from G2 to mitosis [18], and the activation of Akt and Erk, two canonical pathways of cell survival and proliferation, that are regulated by PP2A [19,20]. It is postulated that OA, in sublethal doses, can promote cellular proliferation and activate oncogenic pathways.

## 2. Results and Discussion

### 2.1. OA Generate Cellular Proliferation at Sublethal Doses in Gastric and Colon Epithelium

**Figure 1:**
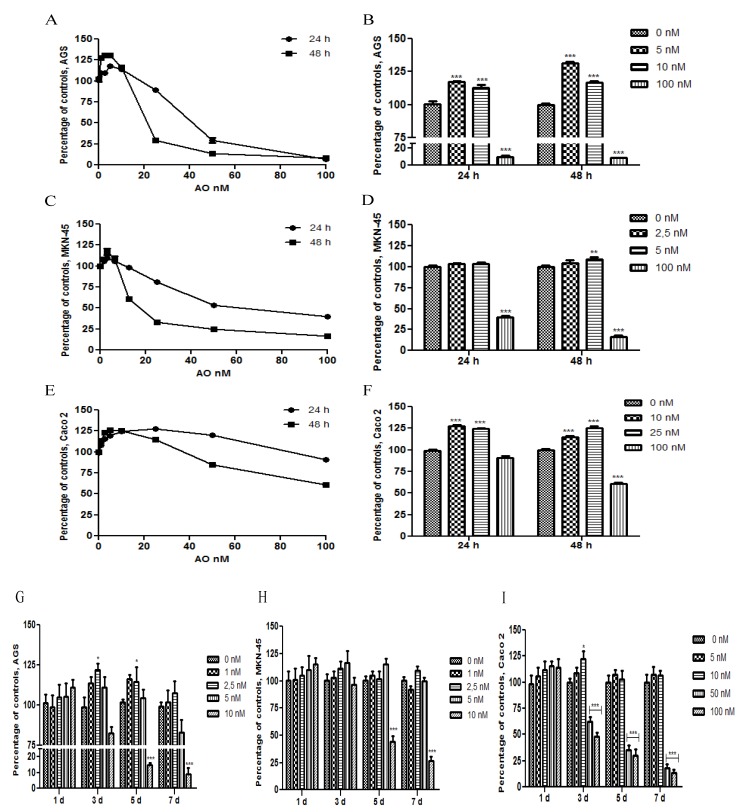
Effect of Okadaic Acid (OA) treatment on cell proliferation. AGS (**A**), MKN-45 (**B**) and Caco 2 (**C**) were incubated for 24 h or 48 h with different concentrations of OA, and the proliferation was measured by AlamarBlue cell viability assay. In **B**, **D**–**F**, the chart bars show significant differences compared to the control (*N* = 12). **G**–**I** show the effect of treatment with OA for 7 days, in AGS, MKN-45 and Caco 2, respectively, using the Trypan blue method exclusion (*N* = 8). * *p* < 0.05; ** *p* < 0.01; *** *p* < 0.005.

The effect of OA on cell proliferation was analyzed in AGS, MNK-45 and Caco 2 cell lines, for 24 h or 48 h. For this, two different methodologies were used: 3-(4,5-dimethylthiazol-2-yl)-2,5-diphenyltetrazolium bromide (MTT) assay and AlamarBlue Cell Viability Assay. No substantial differences were observed between AlamarBlue (Figure 1A,C,E) and MTT assay (data not shown). To display easier cell proliferation to sublethal concentrations, a bar chart with the significant differences is shown (Figure 1B,D,F). To see this effect during longer periods, we used Tripan blue exclusion for seven days (Figure 1G,H,I). Sublethal concentrations showed an increase in the cell proliferation, which in some cases was significant. At concentrations equal to or greater than 10 nM, at longer durations, cells tended to decrease or equate with the control. It was difficult to observe significant differences in this experiment due to the high proliferative rate of these cell lines or to the method limitations. Interestingly, gastric epithelial cells had a similar behavior, but not Caco 2, which showed increased resistance to toxicity of OA, compared with AGS and MKN-45, proving that the cytotoxicity and genotoxicity of OA are cell-line dependent [21].

**Figure 2:**
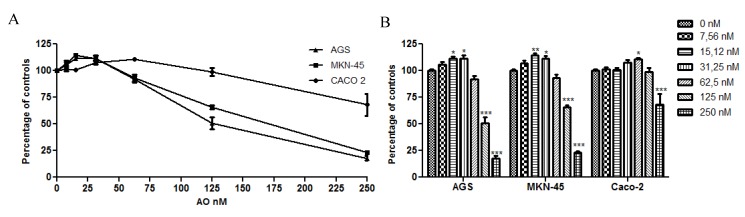
Effect of OA treatment for 4 h on cell viability. The viability was analyzed using the Alamarblue method. The three cell lines show increased resistance to the toxicity of OA with Caco 2 being the most resistant. In **B**, the chart bar shows the significant differences compared to the control (*N* = 12). * *p* < 0.05; ** *p* < 0.01; *** *p* < 0.005.

In order to see the effects of OA on cell proliferation in a more close physiological condition, the cells were exposed for 4 h to the toxin, a point in time at which the stomach presents a complete emptying of the food meal [22], and was analyzed at 48 h. As the with previous results, the cultures showed an increase in cell proliferation at sublethal concentrations, with Caco 2 being more resistant to the toxicity of OA. In fact, the cultures can withstand higher concentrations and remain viable compared with no pulse experiment (Figure 2). These results demonstrate that gastric and colon epithelia can be exposed to lethal levels of OA for short periods of time without activating cellular death, increasing the risk of acting as a tumor promoter.

### 2.2. Overexpression of Cyclin B as an OA Effect

**Figure 3:**
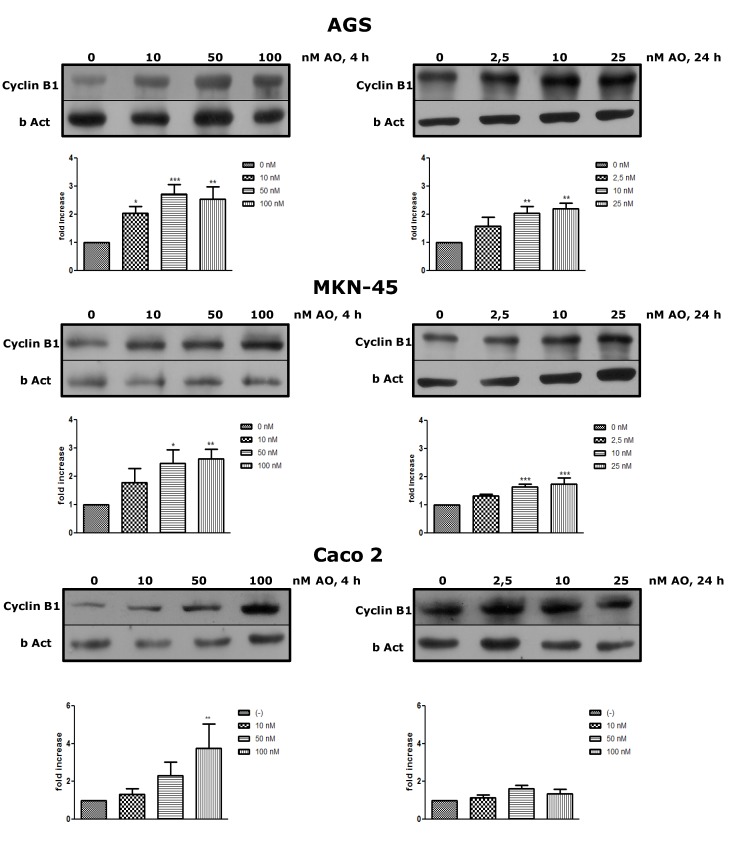
Treatment with OA generates overexpression of Cyclin B. By western blot, an increased expression of Cyclin B after exposure to OA for 4 h or 24 h is shown in all three cell lines. (*N* = 4 for AGS and MKN-45; *N* = 3 for Caco 2). * *p* < 0.05, ** *p* < 0.01, *** *p* < 0.005.

To prove that OA can increase the cell mitotic rate at sublethal concentrations, the expression of Cyclin B was analyzed. After treatment for 4 h or 24 h with OA at different concentrations, Cyclin B increased its expression in the three cells lines, with a dose response effect, even at concentrations in which culture viability was lower (Figure 3). This latter result is interesting since the cells have the input signal to the mitotic cycle; however, deregulation produced by OA could activate apoptosis or other forms of cell death. For the three cell lines, the increase in expression was greater at 4 h than at 24 h, the time at which the cells tend to normalize in respect to the control. The expression of Cyclin B is regulated for multiple pathways, most of them deactivated by PP2A [18]. Therefore, OA would intensify inductive signals of Cyclin B, through inhibition of PP2A.

### 2.3. Activation of Akt and Erk Pathways as an OA Effect

**Figure 4:**
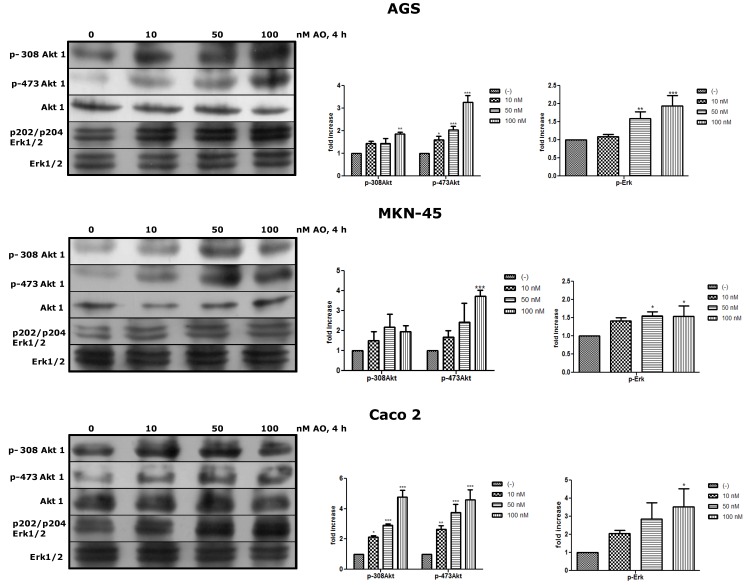
Treatment with OA generates activation of Akt and Erk pathways. By WB, an increased of p308-Akt1, p-473-Akt1 and p-Erk1/2 is shown relative to basal levels of Akt and Erk as an effect of OA after 4 h (*N* = 3). * *p* < 0.05, ** *p* < 0.01, *** *p* < 0.005.

Next, we wanted to see whether OA could activate canonical pathways of proliferation such as Akt and Erk. Akt is the major downstream effector in the PI3-kinase pathway and requires the phosphorylation of Thr308 and Ser473 to be fully active [19]. Erk is central to signaling by growth factors. Their activation involves a cascade of phosphorylation events initiated by stimulation of Ras and ending by MAPK kinases (MEK1/2)-mediated dual phosphorylation of Erk1/2 at Thr and Tyr residues [20]. Both Akt and Erk are essential regulators of cell survival, proliferation, and metabolism and are substrates of PP2A, who acts as deactivator of them. After treatment with OA for 4 h, the three cell lines showed that the phosphorylated forms of Akt and Erk were increased, being significant in some cases (Figure 4).

## 3. Experimental Section

### 3.1. Reagents and Chemicals

Okadaic acid (OA; NRC CRM-OA-c) was purchased from National Research Council (Halifax, Nova Scotia, Canada); Antibodies against Cyclin B1 were obtained from Santa Cruz Biotechnology (Santa Cruz, California, CA, USA); Anti Rabbit IgG Peroxidase conjugate was obtained from Thermo (Rockford, Illinois, IL, USA); Anti Mouse IgG peroxidase conjugate was obtained from Sigma (Saint Louis, Missouri, MO, USA); Erk1 + Erk2, Erk1 + Erk2 (phospho Y204/187 + T202/185), Akt-1, Akt-1 (phospho T308), Akt-1 (phospho S473) and β-Act were obtained from Abcam (Cambridge, Massachusetts, MA, USA). Bovine Serum Albumin (BSA), BCA protein assay, “AlamarBlue cell Viability assay Reagent”, MTT, RIPA Buffer, Halt protease inhibitor cocktail, Phosphatase inhibitor cocktail, BupH Tris-Glycine-SDS Buffer, Surfact-Amp 20, BupH Phosphate Buffered Saline Packs, Nitrocellulose membrane 0.22 µm, Super Signa West Pico Chem iluminescent Substrate and Restore Western blot Stripping Buffer were obtained from Thermo (Rockford, Illinois, IL, USA).

### 3.2. Cell Culture

AGS, MKN-45 and Caco 2 (ATCC) cell lines were cultured in Dulbecco’s Modified Eagle’s Medium (DMEM) containing penicillin (100 μg/mL), streptomycin (100 μg/mL), 1% non-essential amino acids were from Invitrogen (Grand Island, New York, NY, USA), and supplemented with 10% heat-inactivated fetal bovine serum (FBS) were from HyClone (South Logan, Utah, UT, USA). Cells were incubated in 10% CO_2_ at 37 °C and harvested when they reach 70%–90% confluence using 0.25% trypsin/EDTA, from Invitrogen (Grand Island, New York, NY, USA).

### 3.3. Cell Viability Assay

#### 3.3.1. MTT Assay and AlamarBlue Assay

Cells (1 × 10^4^ per well) were cultured in 100 μL of free SFB medium for 24 h. Then the medium was removed and replaced by fresh medium supplemented with OA at different concentrations and incubated at the indicated times. For the MTT assay, cells were incubated with 3-(4,5-dimethylthiazol-2-yl)-2,5-diphenyltetrazolium bromide (MTT), at a final concentration of 0.5 mg/mL for 2 h. Then, 200 μL of 0.1 N HCl-Isopropanol was added, and the plates were agitated for 15 min. The AlamarBlue assay was used according to manufacturer’s recommendations, 10% of reagent was incubated for 2 h. In both assays, optical density of medium in each well was measured with an automatic microplate reader at a test wavelength of 570 nm and a reference wavelength of 690 nm for MTT assay and 600 nm for AlamarBlue assay.

#### 3.3.2. Trypan Blue Assay

Cells (1 × 10^5^) were cultured in 500 μL free SFB medium per well for 24 h. The medium was removed and replaced by fresh medium supplemented with OA at different concentrations and incubated at the indicated times. Cells were detached from the well using 1% trypsin/EDTA, washed once with PBS, and resuspended in 1 mL of medium. Then, cells were counted in a Neubauer chamber, 1:1 with Trypan blue.

The data are expressed considering 100% to the control condition, without OA.

### 3.4. Cell Extracts

For western blot analysis, 3 × 10^5^ cells/ well were seeded in six-well plates in 2 mL of free SFB medium for 24 h. Then, the medium was removed and replaced by fresh medium supplemented with OA at different concentrations and incubated for 4 or 24 h. The cell extracts were prepared removing the medium from plates, washing with cold PBS, and adding RIPA buffer with protease inhibitor and phosphatase inhibitors 1×. After centrifugation (14,000× *g* for 15 min at 4 °C), the protein concentration was determined with BCA protein assay at λ = 595 nm with BSA as standard. Then, the samples were heated for 5 min at 100 °C in the presence of SDS and β-mercaptoethanol.

### 3.5. Western Blotting

Ten micrograms of proteins from total cells were subjected to SDS-PAGE on 15% SDS-polyacrylamide gel electrophoresis and transferred onto nitrocellulose membrane. The membranes were blocked with PBS-buffered saline containing 0.1% Tween-20 (PBST) and 1% BSA for 60 min at room temperature and probed with antibodies against Cyclin B1 (1:2,500), Akt-1 (1:5000), Akt-1 (phospho T308) (1:5000), Akt-1 (phosphor S473) (1:5000), Erk1 + Erk2 (1:5000), Erk1 + Erk2 (phospho Y204/187 + T202/185) (1:5000) and β-actin (1:7500) overnight at 4 °C and washed three times with PBST. The membranes were probed with horseradish peroxidase-conjugated secondary antibodies at 1:5000 for 1 h at room temperature and washed with PBST three times. The immunoblots were visualized by enhanced chemiluminescence. Densitometrical measurement of the band of interest was done using the GelPro 31 software (Media Cybernetics Inc., Silver Spring, MD, USA). Normalization was done using β-actin, Akt-1 or Erk1 + Erk2 values.

### 3.6. Statistical Analyses

The results of the experiments were expressed as the mean ± SE. Comparisons between the groups were made using a two-way ANOVAs and the Bonferroni post-test. The statistical significance was defined as a *P* value smaller than 0.05. The analyses were performed using GraphPad Prism software (La Jolla, California, CA, USA).

## 4. Conclusions

Our results demonstrate that sublethal concentrations of OA can promote the cellular mitotic rate, overexpression of Cyclin B and activation of cell proliferation pathways such as Akt and Erk. In the consumption of shellfish contaminated with diarrheal toxins, the bioavailability of these molecules is very different from the experimental model used. Nevertheless, we believe that our work is a contribution to the elucidation of why these toxins are considered to be tumor promoters.
